# Identification of Novel Mutations in Patients Affected by Gaucher Disease

**DOI:** 10.3390/ijms26083918

**Published:** 2025-04-21

**Authors:** Monia Anania, Miriam Giacomarra, Annalisa D’Errico, Massimo Marano, Immacolata Tartaglione, Marco Spada, Veronica Pagliardini, Maria Rosaria De Paolis, Gaetano Giuffrida, Giulia Duro, Tiziana Di Chiara, Daniele Francofonte, Emanuela Maria Marsana, Paolo Colomba, Giovanni Duro, Carmela Zizzo

**Affiliations:** 1 Institute for Biomedical Research and Innovation (IRIB), National Research Council (CNR), 90146 Palermo, Italy; monia.anania@irib.cnr.it (M.A.); miriam.giacomarra@irib.cnr.it (M.G.); annalisa.derrico@irib.cnr.it (A.D.); daniele.francofonte@irib.cnr.it (D.F.); emanuelamaria.marsana@irib.cnr.it (E.M.M.); paolo.colomba@irib.cnr.it (P.C.); giovanni.duro@irib.cnr.it (G.D.); 2 Unit of Neurology, Neurophysiology, Neurobiology and Psychiatry, Università Campus Bio-Medico di Roma, 00128 Rome, Italy; m.marano@policlinicocampus.it; 3 Fondazione Policlinico Universitario Campus Bio-Medico, Viale Alvaro del Portillo 200, 00128 Rome, Italy; 4 Dipartimento della Donna, del Bambino e di Chirurgia Generale e Specialistica, Università degli Studi della Campania Luigi Vanvitelli, 80138 Napoli, Italy; immacolata.tartaglione@unicampania.it; 5 A.O.U. Città della Salute e della Scienza di Torino—Ospedale Regina Margherita, 10126 Torino, Italy; marco.spada@unito.it (M.S.); vpagliardini@gmail.com (V.P.); 6 Unità Operativa di Ematologia e Trapianto, Polo Oncologico “Giovanni Paolo II”, 73100 Lecce, Italy; depaolis.ematolecce@libero.it; 7 Divisione di Ematologia Sezione Trapianto di Midollo Osseo AOU Policlinico Vittorio Emanuele, 95124 Catania, Italy; gaegiuffrida@gmail.com; 8 Internal Medicine, Ospedale Cattinara, 34149 Trieste, Italy; giulia.duro@libero.it; 9 Excellence Department of Health Promotion, Maternal and Child, Internal and Specialist Medicine “G. D’Alessandro”, University of Palermo, 90127 Palermo, Italy; tiziana.dichiara@unipa.it

**Keywords:** Gaucher disease, glucocerebrosidase, novel mutations, *GBA1*

## Abstract

Gaucher disease is an autosomal recessive disorder caused by dysfunction of the enzyme glucocerebrosidase. The enzyme deficiency is mainly due to mutations in the *GBA1* gene, and it is responsible for the accumulation of glucosylceramide within the lysosomes of monocyte macrophage-derived cells; causing the associated symptomatology. In this paper, we describe six new mutations identified in the *GBA1* gene, which, in combination with other mutations already documented, lead to absent or reduced glucocerebrosidase activity, resulting in pathological accumulation of the specific substrate and the clinical manifestations associated with Gaucher disease. We have identified three mutations (c.1578_1581dup, c.1308dup, and Y492X) that determine the formation of a premature stop codon in the translation process and three missense mutations (C342F, M280L, and Q247R) that lead to amino acid changes in proteins, resulting in decreased glucocerebrosidase activity. These mutations were never observed in our group of healthy control subjects > 1500 individuals. The patients examined had several clinical manifestations, which included hepatosplenomegaly and bone and hematologic involvement; considering the absence of enzyme activity, this suggests that the new mutations described here are associated with type I Gaucher disease. The identification of new mutations in patients with symptoms referable to Gaucher disease increases the molecular knowledge related to the *GBA1* gene and offers to clinicians significant support for the accurate diagnosis of the pathology.

## 1. Introduction

Gaucher disease (GD, OMIM #230800, ORPHA355) is a rare autosomal recessive genetic disorder resulting from mutations in the *GBA1* gene located on chromosome 1 (1q21), which codes for the lysosomal enzyme glucocerebrosidase (GCase or glucosylceramidase or acid β-glucosidase 1 EC: 4.2.1.25) [1,2,3,4,5,6,7]. Within lysosomes, this enzyme plays a crucial role in the hydrolysis of glucosylceramide into ceramide and glucose; when its catalytic activity is impaired, there is an accumulation of glucosylceramide in macrophages, which transform into the Gaucher cells characteristic of the disease. These cells can infiltrate the bone marrow, liver, spleen, and other organs, causing the characteristic symptoms of the pathology [1]. The responsible gene is *GBA1* [8], although less frequently, Gaucher disease can be attributed to mutations in the *prosaposin* gene that codes for an activator (saposin C) of the lysosomal protein β-glucosidase acid [9].

The Gaucher phenotype presents considerable variability, although three main clinical forms can be identified. Type 1 is the most common and generally does not cause neurological damage; it is characterized by the association of hepatosplenomegaly, skeletal changes (osteopenia, bone lesions, and osteonecrosis), and hematologic complications. Type 2, on the other hand, is marked by neurological damage with infantile onset before age two, exhibiting slowed psychomotor development and a rapid course leading to death between the ages of two and four. Type 3, although it has neuronal implications with the onset of symptoms in infancy, has a slower course, allowing survival into the third or fourth decade of life [10,11]. It is important to emphasize that this classification is not rigid, but that Gaucher disease can manifest itself very differently from individual to individual. In fact, the pathology can show heterogeneous signs and symptoms that range from mild to severe and involve increasing problems in the brain and nervous system in all the clinical forms described. Because of this clinical heterogeneity, Gaucher disease can be misdiagnosed with other diseases, causing significant diagnostic delays and serious complications in patients who could have benefited early from enzyme replacement therapy (ERT) or substrate reduction therapy (SRT) [12].

The clinical suspicion of Gaucher disease is advanced on the basis of clinical evidence and family history and is confirmed by biochemical assays, which include the assay of glucocerebrosidase enzyme activity on dried blood spot (DBS), which could be null or reduced [13]. The introduction of specific biomarkers for Gaucher disease significantly improves diagnostic rates, allowing more accurate assessment of the disease severity and treatment efficacy. GD-specific biomarkers improve diagnosis rates of the disease, allowing its severity to be determined and the effectiveness of treatment to be established [14]. Commonly used biomarkers include chitotriosidase and the deacylated metabolite of glucosylceramide, namely glucosyl-sphingosine (Lyso-Gb1) [15,16]. In particular, Lyso-Gb1 represents a direct metabolite of glucocerebrosidase that could play a crucial role in the development of symptoms and disease progression, making it useful as a biomarker, not only in the context of neonatal screening but also to define the severity of the disease at diagnosis and to monitor the clinical course and effectiveness of treatment [17,18]. Genetic analysis, on the other hand, is essential for genotype characterization, genetic counseling, and determination of disease severity, although in an autosomal recessive disease such as Gaucher disease, it could be considered supportive only, since enzymatic analysis should already be discriminating.

This article describes for the first time six mutations (c.1578_1581dup, c.1308dup, Y492X, C342F, M280L, and Q287R) found in biallelic condition in the *GBA1* gene of patients clinically diagnosed with Gaucher disease. The identification of new mutations in patients with symptoms referable to Gaucher increases molecular knowledge of the *GBA1* gene and provides clinicians not only with confirmation but also important support for genotype characterization, genetic counseling, and determination of disease severity. In addition, genetic investigation of the relatives of patients with Gaucher disease allows the identification of pauci- or pre-symptomatic subjects, who, in this way, can start specific therapy as early as possible before organ involvement becomes irreversible, and subjects carrying mutations in the *GBA1* gene, a condition to date considered a risk factor for Parkinson’s disease [19,20].

## 2. Results

In this paper, we describe six new mutations (C342F, M280L, Q247R, c.1578_1581dup, c.1308dup, and Y492X) that we identified in patients with clinical suspicion of Gaucher disease. In the presence of symptoms (in Table 1, we report a summary table comparing phenotypes across all cases) that can be traced back to the disease, we proceeded with a complete diagnostic procedure, including evaluation of glucocerebrosidase enzyme activity, genetic analysis of the *GBA1* gene (the reference sequence used for the nomenclature of the variants is NM_000157.4), and Lyso-Gb1 determination. The genetic alterations, which we found in these patients, are not present in the Human Gene Mutation Database (https://www.hgmd.cf.ac.uk/ac/index.php, accessed on 9 February 2025) accordance with the mutation nomenclature guidelines recommended by the Human Genome Variation Society (https://hgvs-nomenclature.org/stable/, accessed on 9 February 2025).

### 2.1. Case 1: Mutation C342F

The proband of case 1, is a 55-year-old man affected by hepatomegaly, signs of bone fragility, lymphadenopathy, anemia, hyperferritinemia, and MGUS (monoclonal gammopathy of uncertain significance) referable to a diagnosis of Gaucher type I. In addition, the patient had movement disorders attributable to Parkinson’s disease.

We started the diagnostic procedure by performing the enzyme study, which revealed an enzyme activity of 1.8 nmol/h/mL, below the normal reference values for glucocerebrosidase activity (normal reference values: >2.5 nmol/h/mL) and a Lyso-Gb1 value of 11.5 ng/mL (normal reference values: ≤6.8 ng/mL) (Table 2).

**Figure 1:**
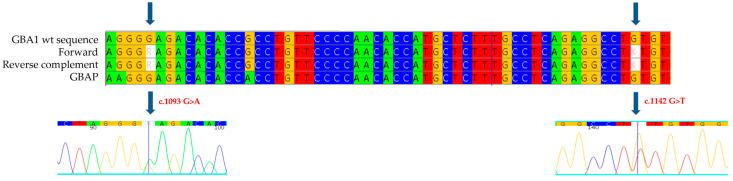
The alignment of forward and reverse respect to GBA1 and GBAP sequences shows the c.1093 G>A and c.1142 G>T mutations to be heterozygous in the proband of case 1. Each nucleotide corresponds to a different color: red for Thymine, blue for Cytosine, yellow for Guanine, green for Adenine. Arrows indicate the mutation. The number present in the electropherograms refers to the position of the nucleotide with respect to the first nucleotide of the amplified sequence.

We then proceeded with the genetic analysis of the eleven exons of the *GBA1* gene and the flanking intronic regions: in exon 8 of the gene, we identified the new missense mutation c.1142 G>T (Figure 1), which causes the substitution of a cysteine with a phenylalanine at position 381 of the protein (p.C381F: C342F-removing signal peptide). This mutation has not been described previously in the literature, and it is not reported in databases of mutations associated with Gaucher disease, but a mutation on the same codon, considered pathogenic and associated with a severe phenotype of Gaucher disease, has been published (c.1141 T>G, p.C381G, also known as C342G-removing signal peptide). In the same exon, we also found a heterozygous mutation, the c.1093 G>A (known in the literature as p.E365K or E326K-removing signal peptide) [21]. This is a variation in a Guanine with an Adenine at position 1093 of the cDNA that results in the replacement of a Glutamic Acid with a Lysine at position 365 of the protein (326 following removal of the signal peptide). The E326K mutation has been reported in databases of mutations associated with Gaucher disease as a mutation of uncertain significance, whether in homozygosity or compound heterozygosity.

The diagnosis of Gaucher disease having been confirmed, the proband of this case was treated with imiglucerase: the first follow-up, performed 6 months after the start of therapy, showed clinical improvement. There was a reduction in Parkinson’s symptoms, a reduction in liver volume, a lowering of hyperferritinemia, and in general, a normalization of hematological values. Lyso-Gb1 accumulation values oscillated over the two years since the start of treatment (between 10.8 ng/mL and 12 ng/mL).

**Figure 2:**
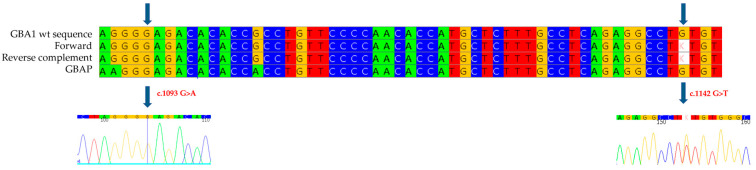
The alignment of forward and reverse respect to GBA1 and GBAP sequences shows only the c.1142 G>T heterozygous mutation as identified in the mother and daughter. Each nucleotide corresponds to a different color: red for Thymine, blue for Cytosine, yellow for Guanine, green for Adenine. Arrows indicate the mutation. The number present in the electropherograms refers to the position of the nucleotide with respect to the first nucleotide of the amplified sequence.

The study was also extended to the proband’s mother and daughter, aged 86 and 22, respectively (Table 2). Both subjects, asymptomatic for Gaucher disease, were studied in order to confirm heterozygous compound mutation in the proband and to provide genetic counseling, since heterozygous mutations in the *GBA1* gene are considered the main risk factor for the development of Parkinson’s disease. In the samples collected from the proband’s mother and daughter, we found normal enzyme activity of 4.5 nmol/h/mL and 3.3 nmol/h/mL, respectively; Lyso-Gb1 values were 7.7 ng/mL and 4.7 ng/mL, respectively. In both cases, only the unknown c.1142 G>T mutation (C342F) was identified (Figure 2), confirming that in the proband, the mutations occur in heterozygous compound mutation, having inherited the E326K mutation from the father (deceased) and the C342F mutation from the mother, who is a carrier. The biallelic condition in the proband is further confirmed by the finding in the daughter of the C342F mutation alone.

### 2.2. Case 2: Mutation M280L

The patient under investigation is a one-year-old child, who presented splenomegaly and thrombocytopenia. The clinical suspicion prompted the diagnostic course for Gaucher disease to be initiated. Analysis of enzyme activity was performed, which showed a greatly reduced value (0.3 nmol/mL/h) (Table 3).

**Figure 3:**
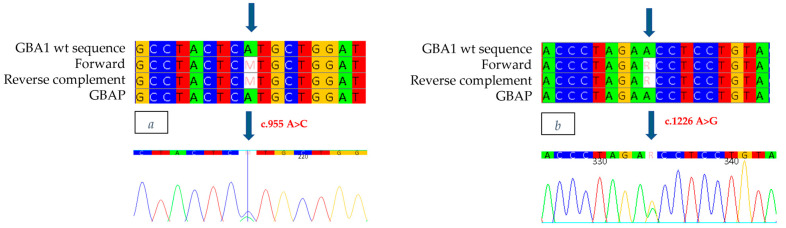
The alignment of forward and reverse respect to GBA1 and GBAP sequences shows the c.955 A>C mutation to be heterozygous in the proband of case 2 (**a**) and the c.1226 A>G mutation to be heterozygous in the proband of case 2 (**b**). Each nucleotide corresponds to a different color: red for Thymine, blue for Cytosine, yellow for Guanine, green for Adenine. Arrows indicate the mutation. The number present in the electropherograms refers to the position of the nucleotide with respect to the first nucleotide of the amplified sequence.

Genetic analysis of the *GBA1* gene revealed, in exon 7, the heterozygous presence of a c.955 A>C missense mutation (Figure 3a), which involves the substitution of an Adenine with a Cytosine at position 955 of the cDNA, thus leading to the substitution of a Methionine with a Leucine at position 319 of the protein (p.M319L: M280L-removing signal peptide). This mutation has never been documented in the literature or in the databases of mutations associated with Gaucher disease. In addition, the c.1226 A>G mutation (Figure 3b), was also identified as heterozygous in exon 9, which causes the replacement of an Asparagine with a Serine at position 409 (p.N409S, also known as N370S-removing signal peptide). The N370S mutation is well known in the literature and is present in databases of Gaucher disease-related mutations, described in patients with the disease in both homozygous and heterozygous compounds [22].

We have no information about the treatment the proband of case 2 underwent.

**Figure 4:**
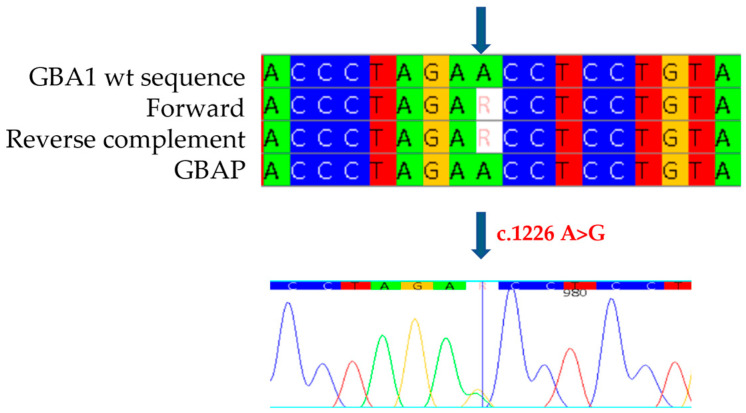
The alignment of forward and reverse respect to GBA1 and GBAP sequences shows only the c.1226 A>G mutation to be heterozygous in the mother. Each nucleotide corresponds to a different color: red for Thymine, blue for Cytosine, yellow for Guanine, green for Adenine. Arrows indicate the mutation. The number present in the electropherogram refers to the position of the nucleotide with respect to the first nucleotide of the amplified sequence.

The study was also extended to the proband’s family members. In the 27-year-old father, who presented with cognitive and growth retardation as well as thrombocytopenia, we found no enzyme activity (0.1 nmol/h/mL/h) and the same mutations identified in the proband. The mother, 31 years old and asymptomatic, showed normal enzyme activity (3.4 nmol/h/mL) and the c.1226 A>G mutation (Figure 4), confirming heterozygous compound mutation in her son. The identification of both M280L/N370S mutations and the absent activity also in the proband’s father prompted us not to limit ourselves to the parents, but to extend the study to the other family members. The paternal grandfather and uncle, aged 65 and 35, respectively, both with mild cognitive and growth retardation, showed slightly reduced enzyme activity (2.0 nmol/h/mL) and only one of the two mutations found in the proband and his father, namely, M280L. In the asymptomatic paternal grandmother, on the other hand, normal enzyme activity was detected together with the N370S mutation, thus confirming the biallelic status of the two M280L/N370S mutations also in the proband’s father. For completeness of the data, we also extended the study to the maternal line, confirming the presence of the N370S mutation as heterozygous in the proband’s grandmother.

### 2.3. Case 3: Mutation Q247R

Patient 3 is an 11-year-old girl who presented with growth retardation, hepatomegaly, splenomegaly, anemia, leucopenia, thrombocytopenia with consequent hemorrhagic diathesis, hyperferritinemia, and subsequent liver transplantation for cholestasis, thus, a general clinical picture that suggested Gaucher disease. Consequently, we started studying the enzymatic activity of glucocerebrosidase, which was found to be 1 nmol/mL/h, below the threshold value of 2.5 nmol/mL/h, resulting in a Lyso-GB1 accumulation value of 218 ng/mL, a pathological value well above the threshold value of 6.8 ng/mL (Table 4).

**Figure 5:**
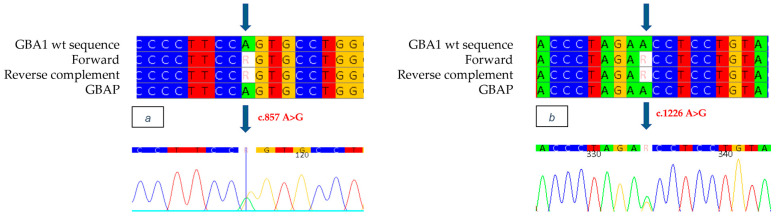
The alignment of forward and reverse respect to GBA1 and GBAP sequences shows the c.857 A>G mutation to be heterozygous in the proband of case 3 (**a**) and the c.1226 A>G mutation to be heterozygous in the proband of case 3 (**b**). Each nucleotide corresponds to a different color: red for Thymine, blue for Cytosine, yellow for Guanine, green for Adenine. Arrows indicate the mutation. The number present in the electropherograms refers to the position of the nucleotide with respect to the first nucleotide of the amplified sequence.

Genetic analysis of the *GBA1* gene allowed the identification as heterozygous of the mutation in exon 7 c.857 A>G (Figure 5a), a variation of an Adenine with a Guanine, which causes the replacement of a Glutamine with an Arginine at position 286 of the protein (p.Q286R: Q247R-removing signal peptide). This mutation, never previously documented in the literature or in databases of mutations associated with Gaucher disease, was identified for the first time in our study. In addition, the known mutation c.1226 A>G was found as heterozygous in exon 9 of the *GBA1* gene. (Figure 5b). Although it was not possible to conduct a family study, the reduced enzyme analysis and the strongly suggestive clinical evidence observed in the patient indicate that the mutations are present in a biallelic condition, thus suggesting that the patient has Gaucher disease.

We have no information about the treatment the proband of case 3 underwent.

### 2.4. Case 4: Mutation c.1578_1581dup

Case 4 is a 45-year-old man with evidence of splenomegaly, signs of bone fragility, anemia, hyperferritinemia, and thrombocytopenia. An accurate anamnesis of the general clinical picture and the exclusion of other possible diagnoses led to an initial hypothesis of Gaucher disease. We then proceeded with the diagnostic protocol that included the analysis of glucocerebrosidase enzyme activity, which was found to be strongly reduced, 1.3 nmol/mL/h, and the determination of Lyso-Gb1, which showed a pathological value of 396 ng/mL (Table 5).

**Figure 6:**
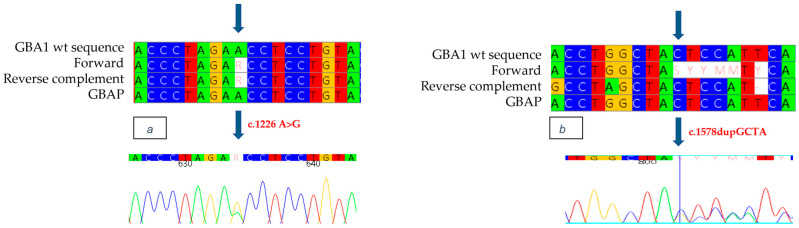
The alignment of forward and reverse respect to GBA1 and GBAP sequences shows the c.1226 A>G mutation to be heterozygous in the proband of case 4 (**a**) and the c.1578_1581dup mutation to be heterozygous in the proband of case 4 (**b**). Each nucleotide corresponds to a different color: red for Thymine, blue for Cytosine, yellow for Guanine, green for Adenine. Arrows indicate the mutation. The number present in the electropherograms refers to the position of the nucleotide with respect to the first nucleotide of the amplified sequence.

The study of the *GBA1* gene allowed the identification of a heterozygous four-nucleotide insertion (c.1578_1581dup) in exon 11 of the gene (Figure 6b), which causes the replacement of tyrosine with a stop codon at position 526 (p.Y526X: Y487X-removing signal peptide) and results in the formation of a truncated, presumably non-functional protein. This mutation, which we describe for the first time, is neither documented in the literature nor present in Gaucher disease databases. Genetic analysis also made it possible to identify the mutation c.1226 A>G as heterozygous in exon 9 (Figure 6a). Although it was not possible to perform a family study, the reduced enzyme analysis and the strongly suggestive clinical evidence observed in the patient indicate that the N370S/c.1578_1581dup mutations are present in a biallelic condition, thus suggesting that the patient has Gaucher disease.

The diagnosis of Gaucher disease having been confirmed, the proband of case 4 has been treated with imiglucerase: the first follow-up, performed 6 months after the start of therapy, showed clinical improvement and a normalization of hematological values. A reduction in splenic volume and a reduction in hyperferritinemia and thrombocytopenia was shown. The Lyso-Gb1 accumulation value also decreased by about 60 ng/mL (from 396 ng/mL to 331 ng/mL).

### 2.5. Case 5: Mutation c.1308dup

Patient 5 is a 17-year-old girl suffering from Gaucher disease, with typical symptoms such as splenomegaly, anemia, leukocytopenia, and hepatomegaly, who came to our attention for monitoring the accumulation product Lyso-Gb1. Our diagnostic pathway, which also included genetic analysis of the *GBA1* gene, allowed us not only to detect a reduced enzyme activity of 0.8 nmol/mL/h and a Lyso-Gb1 value of 31.1 ng/mL (Table 6), compatible with the values observed in a patient undergoing therapeutic treatment, but also to identify a new mutation in the patient’s *GBA1* gene.

**Figure 7:**
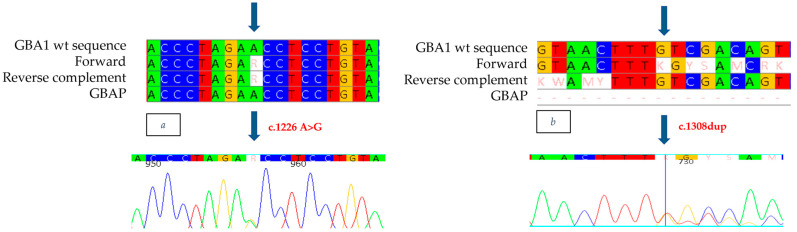
The alignment of forward and reverse respect to GBA1 and GBAP sequences shows the c.1226 A>G mutation to be heterozygous in the proband of case 5 (**a**) and the c.1308dup mutation to be heterozygous in the proband of case 5 (**b**). Each nucleotide corresponds to a different color: red for Thymine, blue for Cytosine, yellow for Guanine, green for Adenine. Arrows indicate the mutation. The number present in the electropherograms refers to the position of the nucleotide with respect to the first nucleotide of the amplified sequence.

The mutation, described for the first time and never previously documented in the literature or Gaucher disease databases, consists of a duplication in exon 9, found to be heterozygous at position 1308 of the cDNA (Figure 7b). This mutation causes a valine to be replaced by a cysteine at position 437 of the protein (398 after removal of the signal peptide), resulting in a frameshift and the formation of a stop codon 32 amino acids downstream from the deleted nucleotide (V437Cfs*32). Genetic analysis also made it possible to identify, in exon 9, the known heterozygous c.1226 A>G mutation (Figure 7a). Although it was not possible to conduct a family study, the reduced enzyme analysis and strongly suggestive clinical evidence observed in the patient indicate that the N370S/c.1308dup mutations are present in a biallelic condition, thus confirming that the patient has Gaucher disease. 

We have no information about the treatment the proband of this case underwent.

### 2.6. Case 6: Mutation Y492X

The patient is a 44-year-old man suffering from Gaucher disease, with anemia, hyperferritinemia, thrombocytopenia, and bone fragility, who came to our attention for monitoring the accumulation product Lyso-Gb1. Our diagnostic pathway, which also included genetic analysis of the *GBA1* gene, allowed us not only to detect a reduced enzyme activity of 0.7 nmol/mL/h and a Lyso-Gb1 value of 18.9 ng/mL (Table 7), compatible with the values observed in a patient undergoing therapeutic treatment, but also to identify a new mutation in the patient’s *GBA1* gene.

**Figure 8:**
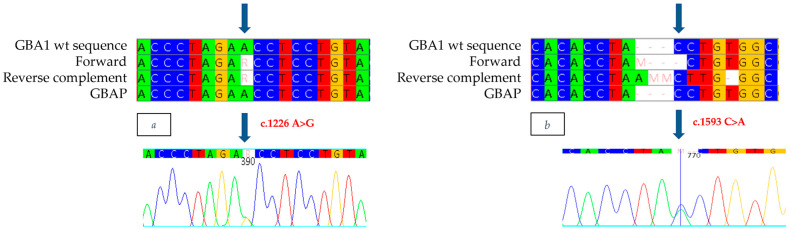
The alignment of forward and reverse respect to GBA1 and GBAP sequences shows the c.1226 A>G mutation to be heterozygous in the proband of case 6 (**a**) and the c.1593 C>A mutation to be heterozygous in the proband of case 6 (**b**). Each nucleotide corresponds to a different color: red for Thymine, blue for Cytosine, yellow for Guanine, green for Adenine. Arrows indicate the mutation. The number present in the electropherograms refers to the position of the nucleotide with respect to the first nucleotide of the amplified sequence.

The new mutation, described for the first time and never previously documented in the literature or the Gaucher disease databases, consists of a nonsense mutation, which is heterozygous, in exon 11 (c.1593 C>A) (Figure 8b). The variation of cytosine with adenine at position 1593 of the cDNA introduces a stop codon at position 531 (p.Y531X:Y492X-removing signal peptide), generating the synthesis of a truncated, non-functional protein (Y492X). Genetic analysis also made it possible to identify, in exon 9, the known mutation c.1226 A>G as heterozygous (Figure 8a). Although it was not possible to conduct a family study, the reduced enzyme analysis and strongly suggestive clinical evidence observed in the patient indicate that the N370S/Y492X mutations are present in a biallelic condition, thus confirming that the patient has Gaucher disease.

The proband of this case came to our laboratories with a diagnosis of Gaucher disease and already on therapy with imiglucerase. In the follow-ups of the next two years, there was almost a normalization of Lyso-Gb1 accumulation values, with an improvement in the clinical and hematological picture.

## 3. Discussion

Early diagnosis of patients suffering from Gaucher disease is of crucial importance in enabling timely therapeutic intervention, and mutation analysis of the *GBA1* gene is a key step in this diagnostic process. Currently, some 721 mutations in the *GBA1* gene associated with Gaucher disease have been identified, including missense, nonsense, small and large deletions/insertions, as well as rearrangements with the GBAP1 pseudogene.

In this study, we have documented six new mutations (C342F, M280L, Q247R, c.1578_1581dup, c.1308dup, Y492X) found in unrelated patients, confirming the heterogeneity already observed in subjects with this disease. The discovery of new genetic variants is essential to expand the database of known mutations and improve genetic diagnosis. Whenever possible, we also extended the genetic and enzymatic analysis to the patients’ families in order to make a significant contribution to understanding the variants. Genetic and enzymatic studies performed on family members are particularly relevant for identifying simple heterozygous individuals (carriers) and heterozygous compound individuals affected by the disease, thus enabling preventive treatment and more rigorous clinical monitoring. Both the insertions (c.1578_1581dup, c.1308dup) and the nonsense mutation (Y492X), together with the missense mutations (C342F, M280L, Q247R) mentioned in this article can be considered responsible for Gaucher disease. This is evidenced by the nature of the mutations themselves and the pathological values of glucocerebrosidase enzyme activity and blood Lyso-Gb1 found in the patients.

**Figure 9:**
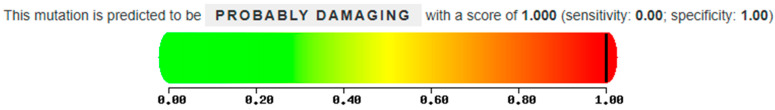
Prediction of the likely malign effect of the C342F mutation.

The proband in case 1 has two heterozygous compound mutations: the known E326K mutation, the significance of which is widely debated but which does not appear to be related to a severe disease phenotype [21], and the new C342F mutation. The latter results from the replacement of cysteine, a polar and hydrophobic amino acid, at position 342 with a phenylalanine, an apolar amino acid, resulting from the removal of the signal peptide. According to an analysis carried out using Polyphen 2 (http://genetics.bwh.harvard.edu/pph2/, accessed on 9 February 2025), a tool for predicting the potential effect of an amino acid substitution on the structure and function of a human protein, we can affirm that the mutation could have a malign effect, with a score of 1 (in a range from 0, indicating benignity, to 1, indicating pathogenicity) (Figure 9).

**Figure 10:**
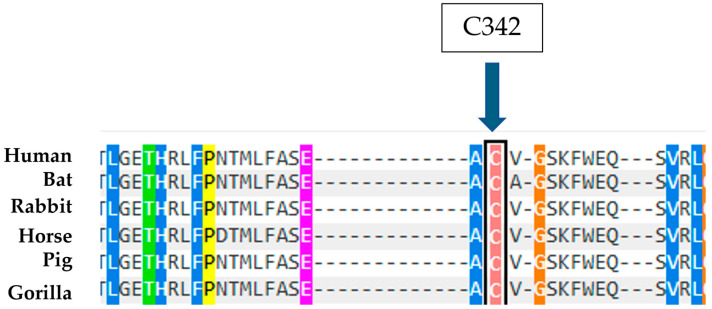
Alignment of human GCase amino acids with sequences from other organisms. The arrow indicates the amino acid at position 342 that was found to be mutated in the case described.

Moreover, it is observed that the amino acid C342 is highly conserved in evolution (Figure 10), a datum supported by the effects of the mutation known in the literature of C342G, which falls on the same codon, is considered pathogenic, and is associated with type II Gaucher disease, an extremely severe phenotype [23]. Therefore, the combination of these predictive data and biochemical results suggest that the C342F in heterozygous compound mutations with a second mutation can be considered causative of Gaucher disease.

In the Supplementary Materials (Figures S1 and S2), we report the molecular structure of the entire protein, with special reference to the p.C381 position.

**Figure 11:**
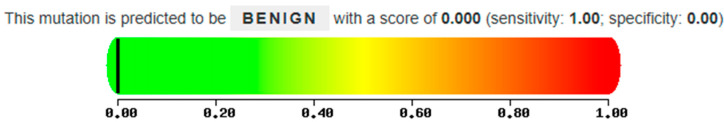
Prediction of the likely harmful effect of the M280L mutation.

**Figure 12:**
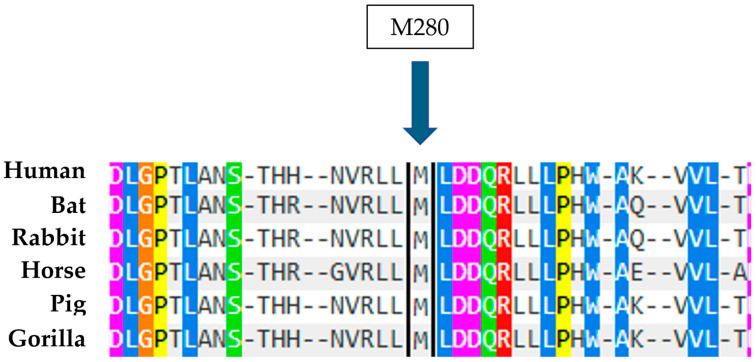
Alignment of human GCase amino acids with sequences from other organisms. The arrow indicates the amino acid at position 280 that was found to be mutated in the case described.

The proband in case 2 has two mutations in a heterozygous compound, the known N370S mutation believed to cause Gaucher disease and the new M280L mutation. This is caused by the replacement of a Methionine, an apolar amino acid, with a Leucine, also an apolar amino acid, at position 280 of the protein following the removal of the signal peptide. In this case, the analysis conducted using Polyphen 2 suggests that the mutation is not directly related to the disease and is therefore potentially benign, as indicated by a score of 0.00 (Figure 11). However, the strong evolutionary conservation of the amino acid M280 (Figure 12) and the absence of glucocerebrosidase activity in both the test subject and the father, both with heterozygous compound for the M280L/N370S mutations, indicate that the M280L mutation could be considered causative of Gaucher disease in heterozygous compound with a second mutation.

**Figure 13:**
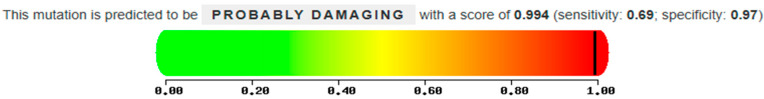
Prediction of the likely harmful effect of the Q247R mutation.

**Figure 14:**
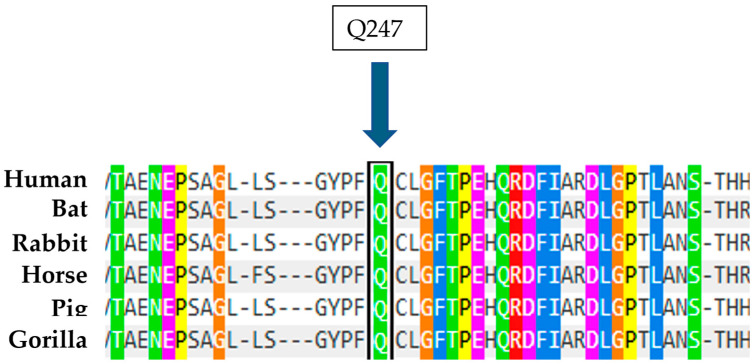
Alignment of human GCase amino acids with sequences from other organisms. The arrow indicates the amino acid at position 247 that was found to be mutated in the case described.

The patient in case 3 has two heterozygous compound mutations: the known mutation N370S and a new mutation Q247R, characterized by the replacement of Glutamine, a polar amino acid, by Arginine, also a polar amino acid, at position 247 of the protein following the removal of the signal peptide. The analysis conducted using the Polyphen 2 tool suggests that this substitution is presumably pathogenic and disease causing, with a score of 0.994 out of a maximum of 1 (Figure 13). Furthermore, a study on the conservation of the amino acid involved, also carried out with Polyphen 2, indicates that Arginine at position 247 is highly conserved in evolution (Figure 14). Therefore, these data, together with the biochemical results showing an enzyme activity of 1 nmol/mL/h and a significant blood accumulation of Lyso-Gb1 of 218 ng/mL, suggest that this mutation, in heterozygous compound with N370S, possesses a high pathogenicity and is therefore causative of Gaucher disease.

For case 4, a mutation was identified in exon 11 of the GBA1 gene in heterozygous compound with the abovementioned N370S; it is a four-nucleotide insertion at position 1578 of the cDNA (c.1578_1581dup), which causes a frameshift and the replacement of a tyrosine with a stop codon at position 526 of the protein. This leads to the formation of a truncated protein containing only 526 amino acids instead of the usual 537, thus probably being non-functional. The presence of observed blood accumulation of Lyso-Gb1, together with the intrinsic nature of the mutation and the clinical signs attributable to the disease, as well as the heterozygous presence of the mutation, considered pathogenic, N370S, suggest the pathogenic role of the c.1578_1581dup.

The mutation in case 5, identified in exon 9 of the gene and associated with the N370S mutation, also consists of an insertion of a base at position 1308 of the cDNA (c.1308dup), which causes the substitution of a valine for a cysteine at position 437 of the protein, resulting in a frameshift and the formation of a premature stop codon. The resulting protein consists of 469 amino acids instead of 537 and is therefore probably non-functional. In this case, the association between the blood accumulation of Lyso-Gb1, the biallelic condition with the N370S mutation, and the nature of the insertion suggests the pathogenicity of the latter.

The patient in case 6 has a cytosine to adenine change at position 1593 of the cDNA, which leads to the formation of the UAA stop codon at that same position, causing premature termination of translation and thus producing a protein composed of 531 amino acids instead of 537. Also in this case, the increased blood level of Lyso-Gb1 and the nature of the mutation itself, associated with N370S, would indicate that this mutation leads to a loss of protein function and, consequently, to the development of the disease.

In accordance with ACMG Standards and Guidelines [24], we have classified the new variants described in this article. The C342F mutation is located within domain 3, which encompasses the active site of the protein (residues 76–381 and 416–430) [25] (PM1) and is absent in control samples (PM2). This represents a novel missense change at an amino acid residue, where a different missense alteration previously classified as pathogenic had been observed [23] (PM5). This missense variant occurs in a gene characterized by a low rate of benign missense mutations, with such variants commonly associated with disease mechanisms (PP2). Additionally, multiple computational evidences, for example, Polyphen 2, UniProt, and Alphafold, support the hypothesis of a deleterious effect on the gene or its product (PP3), and the patient’s phenotype is highly specific to Gaucher disease, which is defined by a singular genetic etiology (PP4). In light of these pathogenicity criteria and considering the presence of three more moderate evidence indicators supporting pathogenicity, as outlined in the ACMG Standards and Guidelines, this mutation can be regarded as Likely Pathogenic (Table 8).

The M280L mutation meets the pathogenicity criteria PM1, PM2, PP2, and PP4. Additionally, it has been identified in trans with a pathogenic variant (PM3) and has demonstrated co-segregation with Gaucher disease in multiple family members, as evidenced in this study (PP1). Based on these pathogenicity criteria and having three moderate evidence of pathogenicity indicators, the mutation can be classified as Likely Pathogenic (Table 8).

The Q247R mutation meets the pathogenicity criteria PM1, PM2, PM3, PP2, PP3, and PP4. Given these pathogenicity criteria and the presence of three moderate evidence for pathogenicity indicators, this mutation can be classified as Likely Pathogenic (see Table 8).

c.1578_1581 dup results in a frameshift in the GBA1 gene, where loss of function (LOF) is a recognized mechanism of disease [26,27] (PVS1). It causes variations in protein length due to in-frame insertions in a non-repetitive region (PM4). Additionally, it meets the pathogenicity criteria PM2, PM3, and PP4. Based on these pathogenicity criteria, with one Very Strong (PVS1) and more than two moderate evidence indicators of pathogenicity, this mutation can be classified as Pathogenic (Table 8).

c.1308dup also satisfies the pathogenicity criteria PVS1, PM1, PM2, PM3, PM4, PM5 [28], and PP4. Given these pathogenicity criteria and having one Very Strong (PVS1) with more than two moderate evidence indicators of pathogenicity, this mutation can be considered Pathogenic (Table 8).

The Y492X mutation meets the pathogenicity criteria PVS1, PM2, PM3, PM4, and PP4. Based on these pathogenicity criteria, with 1 Very Strong (PVS1) and more than 2 Moderate evidence of pathogenicity indicators, the mutation can be classified as Pathogenic (Table 8).

**Figure 15:**
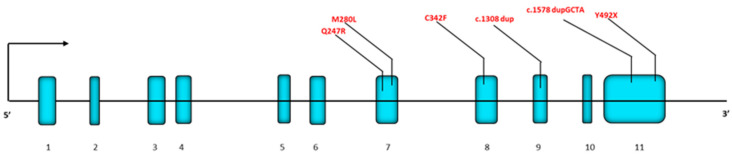
Diagram of the GBA1 gene with the new mutations reported in this paper.

In Figure 15, we report a schematic diagram of the *GBA1* gene showing the location of all new mutations reported in this paper.

All the new mutations described in this article, associated with already known causative mutations, lead to a decrease in enzyme activity and a blood accumulation of Lyso-Gb1, thus contributing to the characteristic clinical presentation of Gaucher disease. These clinical pictures complicate the identification of Gaucher patients due to the marked heterogeneity between genotype and phenotype, which leads to delays in diagnosis and, consequently, to a delay in starting therapy. All patients analyzed in this study presented symptoms attributable to Gaucher disease independently of the traditional classification into the three disease types, the boundaries of which are nuanced and therefore do not allow a clear-cut categorization.

## 4. Materials and Methods

### 4.1. Patients

Peripheral blood was collected using EDTA as an anticoagulant and dried on absorbent paper (dried blood spot, DBS). Genetic and enzymatic studies were performed at the Centre for Research and Diagnosis of Lysosomal Storage Disorders of IRIB-CNR in Palermo and were approved by the Hospital Ethics Committee of the University of Palermo. Signed informed consent was obtained from the patients. We studied 1500 subjects who arrived in our laboratories with clinical suspicion of Gaucher disease, aged 15 to 80 years, both male and female. The cohort of 1500 subjects was genetically screened for each specific region, and we found these new mutations in 6 of them, the probands, and from these, we investigated family members.

### 4.2. Glucocerebrosidase Activity Assay

Glucocerebrosidase analysis was determined by the dried blood filter paper test (DBFP) described by Chamoles et al. [29], with modifications. For the GCase enzymatic assay, the artificial substrate 4-methylumbelliferyl β-F-glucopyranoside (SIGMA-11.6 mmol/L) in Phosphate–Citrate Buffer (CPB, pH 5.2), specific for GCase, conjugated with a fluorophore was used. The fluorescence values detected by the fluorometer (λex: 365 nm, λem: 448 nm) reflect the quantity of substrate degraded by the enzyme present in the analyzed sample. Fluorescence data were processed by an algorithm that provided for each sample an enzymatic activity value expressed in nmol/h/mL. Furthermore, we added to the reaction mixture a cell lysis solution at pH 5.2 (CPB, TRITON-X 100, SODIUM-TAUROCHOLATE) and CBE (Conduritol B epoxide), a specific inhibitor for the non-lysosomal β-glucosidase (NLGCase), to discriminate between GCase and NLGCase. Samples with the reaction mixture were incubated for 18 h at 37 °C in a thermomixer (900 RPM) covered with film.

### 4.3. DNA Extraction

Genomic DNA was isolated from dried blood spot using a Qiagen (Milano, Italy) EZ1 advanced XL automatic extractor, and an EZ1 Advanced XL DNA investigator card (a programmed card containing protocols for DNA extraction by filter paper) was used in combination with the EZ1 DNA investigator kit. DNA concentrations were estimated using a biophotometer (Eppendorf, Hamburg, Germany).

### 4.4. PCR and Sequencing

The search for mutations in the *GBA1* gene was performed by Sanger sequencing. Two pairs of PCR primers were designed to analyze exclusively *GBA1* and identify recombinant alleles with GBAP at 0.4 μM concentration in the reaction mixture. Long PCR amplification of the two macroregions, exon1–intron 5 and intron5–exon 11 (two reaction mixtures for each sample), was performed using biotechrabbit™ Long-Range PCR Master Mix (Voden Medical Life Science and Diagnostic Division, Meda, MB, Italy), 2X, following the basic protocol and cycling program in the reaction manual. PCR Enhancer and MgCl_2_ were not supplied in the mixture. The template DNA concentration was 50–100 ng in each reaction mixture. Exon 1–intron 5 and intron5–exon 11 PCR products were purified by ExoSAP-IT™ PCR and used for sequencing the specific 1–5 exons and 6–11 exons of GBA1. Eurofins Genomics service (Vimodorne, MI, Italy) performed Sanger sequencing. Sequence analysis was performed using LI-COR Align IR (Licoln, NE, USA) and Chromas bioinformatic programs 2.5.

### 4.5. Glucosylsphingosine Assay

Detection of glucosylsphingosine (Lyso-Gb1) was performed in dried blood spots using the LC-MS/MS technique [30,31,32,33].

## 5. Conclusions

Clinically, Gaucher disease should be considered in all patients with symptoms attributable to the disease, an uncertain diagnosis, and an atypical clinical course or a clinical picture with an unclear systemic implication. This clinical approach would help limit misdiagnosis between Gaucher disease and other diseases or cases of missed diagnosis. This could help reduce the time between the onset of symptoms and the diagnosis of Gaucher disease, avoiding unnecessary treatments for patients and initiating the available therapy specific to the disease from which they suffer.

The results described here suggest that these six new mutations may be correlated with the manifestations of Gaucher disease. These new data can help the clinician in the diagnosis of the disease and increase clinical and molecular knowledge in the correlation between mutations in the *GBA1* gene and the disease phenotype.

## Figures and Tables

**Table 1:** Summary table comparing phenotypes across all cases. “-” indicates absence of the symptom; “+” indicated presence of the symptom.

	Proband Case 1	Proband Case 2	Proband Case 3	Proband Case 4	Proband Case 5	Proband Case 6
**Anemia**	+	-	+	+	+	+
**Thrombocytopenia**	-	+	+	+	-	+
**Hepatomegaly**	+	-	+	-	+	-
**Bone fragility**	+	-	-	+	-	+
**Lymphadenopathy**	+	-	-	-	-	-
**Hyperferritinemia**	+	-	+	+	-	+
**MGUS**	+	-	-	-	-	-
**Parkinsonism**	+	-	-	-	-	-
**Splenomegaly**	-	+	+	+	+	-
**Growth retardation**	-	-	+	-	-	-
**Leukocytopenia**	-	-	+	-	+	-
**Hemorrhagic dathesis**	-	-	+	-	-	-
**Liver transplant**	-	-	+	-	-	-

**Table 2:** Molecular and clinical data of patients with the C342F mutation. The values in bold are patological.

Patient	Sex	Age	Mutation	GCase Activity (nmol/mL/h) Normal Range > 2.5 nmol/mL/h	Lyso-Gb1 in Blood (ng/mL) Normal Range < 6.8 ng/mL	Clinical Signs
Proband of case 1	M	55	E326K/C342F	**1.8**	**11.5**	Hepatomegaly, bone fragility, lymphadenopathy, anemia, hyperferritinemia, MGUS, Parkinsonism
Mother	F	89	C342F	4.5	**7.7**	No info
Daughter	F	22	C342F	3.3	4.7	No info

**Table 3:** Molecular and clinical data of patients with the M280L mutation. The values in bold are patological.

Patient	Sex	Age	Mutation	GCase Activity (nmol/mL/h) Normal Range > 2.5 nmol/mL/h	Lyso-Gb1 in Blood (ng/mL) Normal Range < 6.8 ng/mL	Clinical Signs
Proband of case 2	M	1	M280L/N370S	**0.3**	Not performed	Splenomegaly, thrombocytopenia
Father	M	27	M280L/N370S	**0.1**	Not performed	Growth retardation, thrombocytopenia
Grandfather paternal	M	65	M280L/wt	2.0	Not performed	No info
Paternal Uncle	M	35	M280L/wt	2.0	Not performed	No info
Grandmother paternal	F	61	wt/N370S	2.4	Not performed	No info
Mother	F	31	wt/N370S	3.4	Not performed	No info
Grandmother maternal	F	56	wt/N370S	3.9	Not performed	No info
Grandfather maternal	M	61	wt/wt	6.8	Not performed	No info

**Table 4:** Molecular and clinical data of patient with the Q247R mutation. The values in bold are patological.

Patient	Sex	Age	Mutation	GCase Activity (nmol/mL/h) Normal Range > 2.5 nmol/mL/h	Lyso-Gb1 in Blood (ng/mL) Normal Range < 6.8 ng/mL	Clinical Signs
Proband of case 3	F	11	N370S/Q247R	**1.0**	**218.0**	Growth retardation, hepatomegaly, splenomegaly, anemia, leukocytopenia, thrombocytopenia, hyperferritinemia, hemorrhagic diathesis, liver transplant for cholestasis

**Table 5:** Molecular and clinical data of patient with the c.1578_1581dup mutation. The values in bold are patological.

Patient	Sex	Age	Mutation	GCase Activity (nmol/mL/h) Normal Range > 2.5 nmol/mL/h	Lyso-Gb1 in Blood (ng/mL) Normal Range < 6.8 ng/mL	Clinical Signs
Proband of case 4	M	45	N370S/c.1578_1581dup	**1.3**	**396**	Splenomegaly, bone fragility, anemia, hyperferritinemia, thrombocytopenia

**Table 6:** Molecular and clinical data of patient with the c.1308dup mutation. The values in bold are patological.

Patient	Sex	Age	Mutation	GCase Activity (nmol/mL/h) Normal Range > 2.5 nmol/mL/h	Lyso-Gb1 in Blood (ng/mL) Normal Range < 6.8 ng/mL	Clinical Signs
Proband of case 5	F	17	N370S/c.1308dup	**0.8**	**31.1**	Splenomegaly, anemia, leukocytopenia, hepatomegaly

**Table 7:** Molecular and clinical data of patient with the Y492X mutation. The values in bold are patological.

Patient	Sex	Age	Mutation	GCase Activity (nmol/mL/h) Normal Range > 2.5 nmol/mL/h	Lyso-Gb1 in Blood (ng/mL) Normal Range < 6.8 ng/mL	Clinical Signs
Proband of case 6	M	44	N370S/Y492X	**0.7**	**18.9**	Anemia, hyperferritinemia, thrombocytopenia, bone fragility

**Table 8:** Classifications ACMG of the six new variants described in this article.

Mutation	Criteria for Classifying Pathogenic Variants	ACMG Classification
C342F	PM1, PM2, PM5, PP2, PP3, PP4	Likely Pathogenic
M280L	PM1, PM2, PM3, PP1, PP2, PP4	Likely Pathogenic
Q247R	PM1, PM2, PM3, PP2, PP3, PP4	Likely Pathogenic
c.1578_1581dup	PVS1, PM2, PM3, PM4, PP4	Pathogenic
c.1308dup	PVS1, PM1, PM2, PM3, PM4, PM5, PP4	Pathogenic
Y492X	PVS1, PM2, PM3, PM4, PP4	Pathogenic

## Data Availability

The raw data supporting the conclusions of this article will be made available by the authors on request.

## Supplementary Materials

The supporting information can be downloaded at: https://www.mdpi.com/article/10.3390/ijms26083918/s1.
