# Supplementary material for: Identification of Novel Mutations in Patients Affected by Gaucher Disease
Source: Int J Mol Sci. 2025 Apr 21;26(8):3918. doi: 10.3390/ijms26083918 (PMC12028185; doi:10.3390/ijms26083918)
Supplement: Supplementary file 1 [file ijms-26-03918-s001.zip › ijms-3496132-supplementary.pdf]

PROTEIN STRUCTURAL ANALYSIS

a.a.	benign	ambiguous	pathogenic	mean
C381		1:S	5:F,G,R,W,Y	0.844

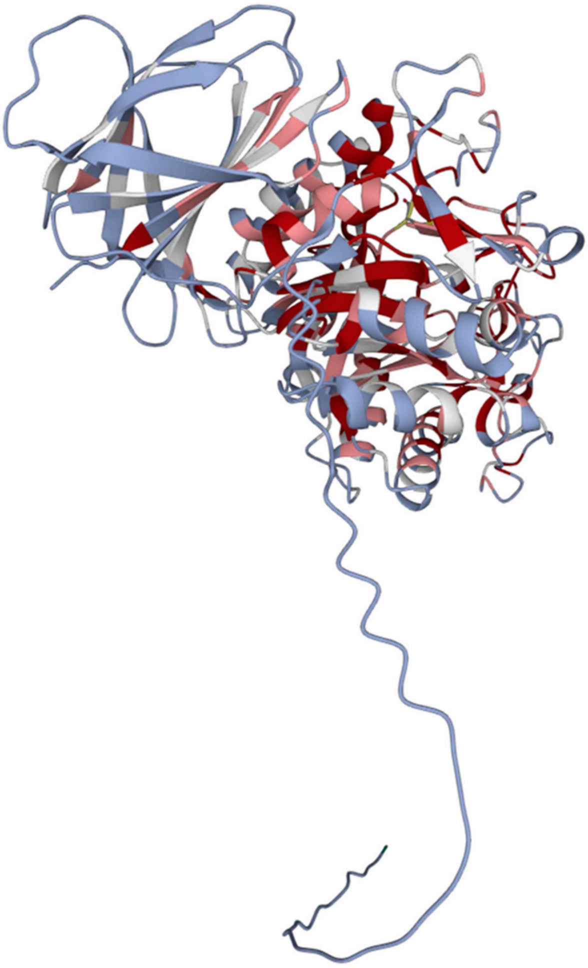

Supplemental Figure S1. Protein structure of the enzyme glucocerebrosidase.

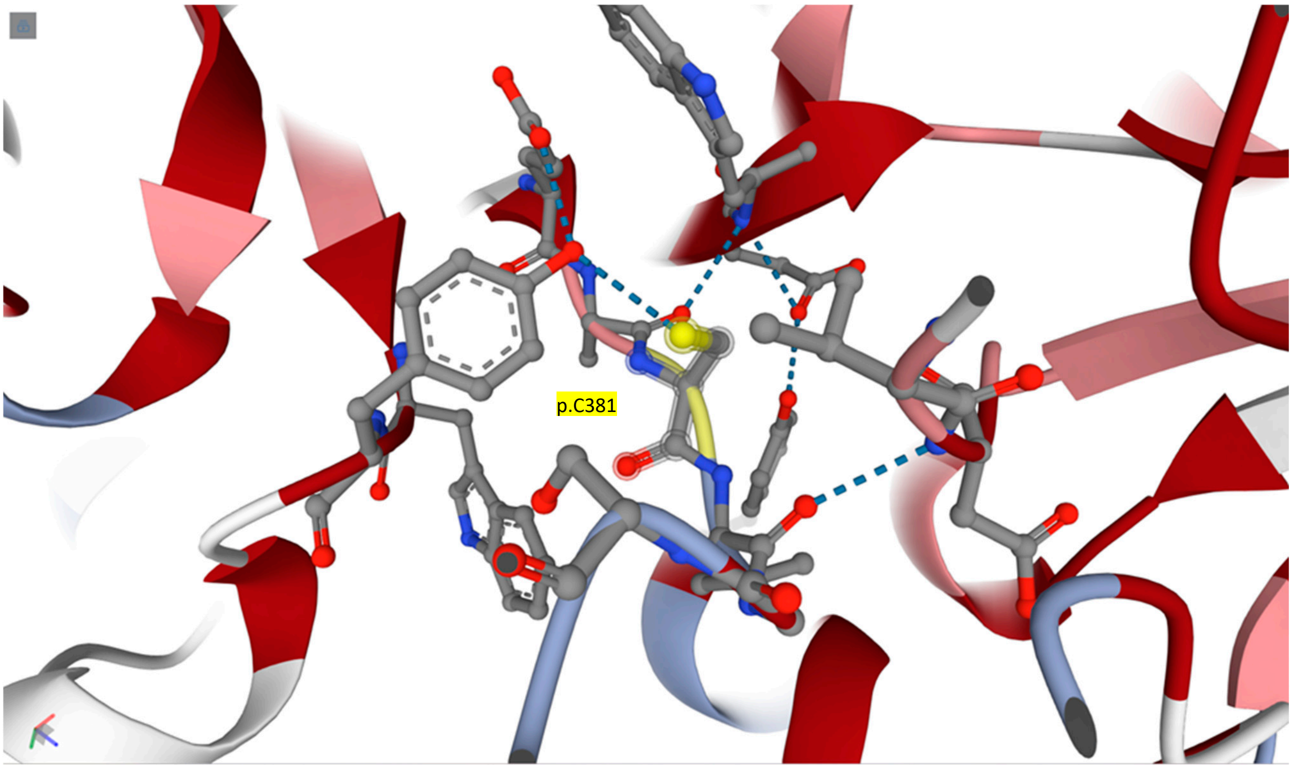

Supplemental Figure S2. Structure of glucocerebrosidase: p.C381 position is highlighted in yellow (which becomes C342- removing signal peptide): this is the amino acide involved in the first case.
